# Case Report: Getting a Peek at the Angle of a Patient with Severe Keratoconus

**DOI:** 10.3389/fopht.2022.843224

**Published:** 2022-03-02

**Authors:** Rumi Kawashima, Kenji Matsushita, Kazuhiko Ohnuma, Naoyuki Maeda, Shizuka Koh, Kohji Nishida

**Affiliations:** ^1^ Department of Ophthalmology, Osaka University Graduate School of Medicine, Suita, Japan; ^2^ Laboratorio de Lente Verde, Chiba, Japan; ^3^ Integrated Frontier Research for Medical Science Division, Institute for Open and Transdisciplinary Research Initiatives, Osaka University, Suita, Japan

**Keywords:** keratoconus, glaucoma, critical angle, RetCam 3, angle - section

## Abstract

Visualization of the iridocorneal angle, which contains the aqueous humor circulatory system and controls intraocular pressure, is important for diagnosing and managing glaucoma; however, the presence of keratoconus, keratoglobus, or severe myopia may enable direct angle visualization without gonioscopy contact lenses or applying a coupling gel. We present the first report of a case in which the iridocorneal angle was viewed directly in an eye with keratoconus using the RetCam without applying gel to the cornea. This method overcame the inability to view the angle directly in a normal eye because of the total internal reflection.

## Case Report

**Figure 1:**
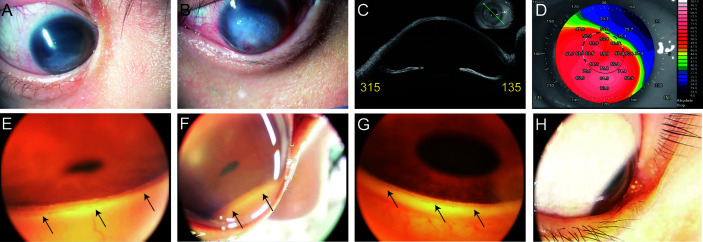
Photographs of the anterior eye: **(A)** in right eye, **(B)** in left eye. Images of the anterior segment optical coherence tomography (CASIA SS-2000, Tomey Corporation, Nagoya, Aichi, Japan) in right eye: **(C)** the cross-sectional image, **(D)** keratometric power map. An image of the angle in the inferior quadrant using the RetCam: **(E)** with gel on the cornea of an eye with severe keratoconus, **(F)** without gel on the cornea of an eye with severe keratoconus, **(G)** with gel on the cornea of a control eye, and **(H)** without gel on the cornea of a control eye. The black arrows indicate the AC angle.

A 30-year-old woman with Down syndrome had been followed for keratoconus and allergic conjunctivitis. Slit-lamp examination showed a corneal scar following acute corneal hydrops in the right eye and active acute corneal hydrops in the left eye (**Figures 1A, B**). She was treated with steroid eye drops for allergic conjunctivitis for many years. So, she was suspected to have steroid-induced glaucoma and has been treated with glaucoma eyedrops and oral medication. We were not able to measure corneal curvature by using a keratometry because of corneal opacity and poor fixation, but we managed to measure a topography by using anterior segment optical coherence tomography (OCT), (CASIA SS-2000, Tomey Corporation, Nagoya, Aichi, Japan) in her right eye (**Figures 1C, D**). Although her vision was worsening and her glaucoma presumably progressing, the intellectual disability associated with Down syndrome made ophthalmologic assessment, i.e., measurement of intraocular pressure (IOP), gonioscopy, and biometric measurement, difficult. Therefore, these examinations were performed with the patient under general anesthesia.

The IOP was 28 mmHg in the right eye and 36 mmHg in the left eye using a TonoPen XL (Mentor, Santa Barbara, CA, USA) under sedation. The corneal diameters were 11×11 mm bilaterally. A-mode ultrasonography showed axial lengths of 30.88 mm in the right eye and 30.9 mm in the left eye. Ultrasound biomicroscopy (UBM) showed open angles, no abnormal anterior chamber (AC) angles, and no peripheral anterior synechia bilaterally. The fundus could not be visualized because of the corneal opacity, but B-mode ultrasonography did not show an ocular abnormalities. Finally, using the RetCam 3 Ophthalmic Imaging System (Clarity Medical Systems, Punjab, India), we observed the AC angle by applying coupling gel to the cornea of the right eye (**Figure 1E**) as reported previously (1). Briefly, we placed the 130° lens probe on the patient’s eye with a coupling gel. The probe was positioned at the limbus opposite to the angle being photographed. We could not clearly record the angle in the left eye because of severe corneal opacity. At that time, we found that we could directly visualize the gonio-angle in the inferior quadrant of the right eye without applying gel to the cornea (**Figure 1F**). We performed an ab interno microhook trabeculotomy in the right eye and ab externo trabeculotomy in the left eye because of the corneal opacity. One year after the glaucoma surgery, the ocular status remained good and the IOP is within the normal range with glaucoma eyedrops.

## Discussion

**Figure 2:**
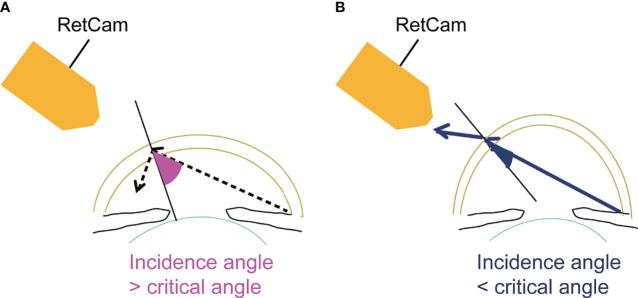
Schema of light from the AC angle **(A)** in a normal eye and **(B)** in an eye with keratoconus.

Assessing the iridocorneal angle is essential for diagnosing and managing glaucoma. Various imaging techniques have been developed to accomplish this, i.e., optical coherence tomography (OCT), UBM, gonioscopic photography, and the RetCam (2). Optical tomographic imaging techniques include OCT and UBM. OCT is a non-contact and non-invasive imaging technique that facilitates fast and easy estimation of the AC. However, we cannot visualize the AC angle directly and evaluate it in detail. UBM can visualize structures of the iridocorneal angle and ciliary body in more detail; however, the transducer must be in contact with the eye, which induces patient discomfort, and the examiner must be experienced. With photographic methods, the iridocorneal region is captured using a camera. These methods include gonioscopic photography and the RetCam. Gonioscopy is the fundamental and most important examination for diagnosing and managing glaucoma. However, it is impossible to directly view the angle in a normal eye because of the total internal reflection (3). Trantas initially reported examination of the angle in 1907 by scleral indentation using his finger (4). Second, Mizuo examined the inferior angle in patients by everting the lower lid and filling the cul-de-sac with saline (5). Salzmann recognized that the normal angle was invisible because of the total internal reflection (6) and viewed the angle through a contact lens at first (7). At present, we usually use gonioscopy contact lenses to overcome the total internal reflection. Gonioscopy requires effort to examine the eye and causes patient discomfort (8) but allows direct observation of the angle structures cost-effectively and in detail. The direct imaging of the angle is effective to diagnose, manage, and treat glaucoma by assessing the detailed information on angle anatomy. The RetCam was developed originally to obtain widefield and high-resolution photographs of the pediatric fundus (9) but also can image the AC angle as identified by direct gonioscopy (10). For angle imaging, the patient is required to be supine, a coupling gel is placed between the patient’s cornea and the camera lens, and the AC angle opposite to the lens can be viewed (**Figure 1G**). The gel functions as an optical interface to eliminate the total internal reflection at the corneal tear film-air interface (2). In most eyes, the angle cannot be visualized without applying gel to the cornea (**Figure 1H**). However, if the light from the angle of the AC strikes the cornea at an angle steeper than 46° (the critical angle), the light will exit the eye and the angle will be visible. This can occur rarely in eyes with keratoconus, keratoglobus, or severe myopia (3) (**Figure 2**). This observation technique could be also applied to a slit-lamp microscopy. We can estimate the critical angle by using anterior segment OCT, but it is the further study to develop the way to identify patients who are suitable for this imaging technique.

This is the first report to successfully record a direct and non-contact view of the iridocorneal angle in an eye with severe keratoconus using the RetCam without the need to use a material such as gel on the cornea.

## Data Availability Statement

The original contributions presented in the study are included in the article/supplementary material. Further inquiries can be directed to the corresponding author.

## Ethics Statement

The patient provided her written informed consent to publish of this case report, including all data and images.

## Author Contributions

KM, NM, and KO contributed to conception and design of the study. KO and RK acquired the data and performed the analysis. RK and KM wrote the draft of the manuscript. RK, KM, KO, NM, SK, and KN contributed to manuscript revision and approved the submitted version and take responsibility for the integrity of the data and the accuracy of the data analysis.

## Funding

Research supported by JSPS KAKENHI grants 26462686, 18K09406 (KM), 20K18379 (RK)

## Conflict of Interest

The authors declare that the research was conducted in the absence of any commercial or financial relationships that could be construed as a potential conflict of interest.

## Publisher’s Note

All claims expressed in this article are solely those of the authors and do not necessarily represent those of their affiliated organizations, or those of the publisher, the editors and the reviewers. Any product that may be evaluated in this article, or claim that may be made by its manufacturer, is not guaranteed or endorsed by the publisher.
